# Occurrence and genetic characteristics of *Cryptosporidium* spp. and *Enterocytozoon bieneusi* in pet red squirrels (*Sciurus vulgaris*) in China

**DOI:** 10.1038/s41598-020-57896-w

**Published:** 2020-01-23

**Authors:** Lei Deng, Yijun Chai, Run Luo, Leli Yang, Jingxin Yao, Zhijun Zhong, Wuyou Wang, Leiqiong Xiang, Hualin Fu, Haifeng Liu, Ziyao Zhou, Chanjuan Yue, Weigang Chen, Guangneng Peng

**Affiliations:** 1 The Key Laboratory of Animal Disease and Human Health of Sichuan Province, College of Veterinary Medicine, Sichuan Agricultural University, Chengdu, Sichuan 611130 China; 2 Chengdu Research Base of Giant Panda Breeding, Sichuan Key Laboratory of Conservation Biology for Endangered Wildlife, Sichuan Academy of Giant Panda, Chengdu, Sichuan Province 611130 China

**Keywords:** Parasite biology, Parasite development

## Abstract

*Cryptosporidium* spp. and Enterocytozoon bieneusi are two well-known protist pathogens which can result in diarrhea in humans and animals. To examine the occurrence and genetic characteristics of *Cryptosporidium* spp. and E. bieneusi in pet red squirrels (Sciurus vulgaris), 314 fecal specimens were collected from red squirrels from four pet shops and owners in Sichuan province, China. *Cryptosporidium* spp. and E. bieneusi were examined by nested PCR targeting the partial small subunit rRNA (SSU rRNA) gene and the ribosomal internal transcribed spacer (ITS) gene respectively. The infection rates were 8.6% (27/314) for *Cryptosporidium* spp. and 19.4% (61/314) for E. bieneusi. Five *Cryptosporidium* species/genotypes were identified by DNA sequence analysis: *Cryptosporidium* rat genotype II (n = 8), *Cryptosporidium* ferret genotype (n = 8), *Cryptosporidium* chipmunk genotype III (n = 5), *Cryptosporidium* rat genotype I (n = 4), and *Cryptosporidium* parvum (n = 2). Additionally, a total of five E. bieneusi genotypes were revealed, including three known genotypes (D, SCC-2, and SCC-3) and two novel genotypes (RS01 and RS02). Phylogenetic analysis revealed that genotype D fell into group 1, whereas the remaining genotypes clustered into group 10. To our knowledge, this is the first study to report *Cryptosporidium* spp. and E. bieneusi in pet red squirrels in China. Moreover, C. parvum and genotype D of E. bieneusi, previously identified in humans, were also found in red squirrels, suggesting that red squirrels may give rise to cryptosporidiosis and microsporidiosis in humans through zoonotic transmissions. These results provide preliminary reference data for monitoring *Cryptosporidium* spp. and E. bieneusi infections in pet red squirrels and humans.

## Introduction

*Cryptosporidium* spp. and *Enterocytozoon bieneusi*, causative agents of cryptosporidiosis and microsporidiosis, are two important opportunistic intestinal pathogens that can infect vertebrate and invertebrate, posing a significant threat to public health^1^. Humans are infected with these pathogens mainly via the fecal-oral route with anthroponotic and zoonotic transmission or via food-borne and water-borne transmission^2,3^. Clinical manifestations of infection with these pathogens are often inconsistent due to the variabilities in the health condition of infected hosts^4,5^. In healthy individuals, these pathogens usually cause asymptomatic infection or self-limiting diarrhea^6^. However, infection may also result in chronic or life-threatening diarrhea in immunocompromised individuals, such as patients with acquired immunodeficiency syndrome and patients who had undergone organ transplantation^7^. In addition to humans, there are a variety of animals that can act as hosts for these two pathogens, including various mammals, reptiles, birds, amphibians and insects^8^. Therefore, *Cryptosporidium* spp. and *E. bieneusi* have been recognized as category B pathogens by the National Institutes of Health due to their ease of transmission in spite of low mortality^9^.

To detect and evaluate potential zoonotic transmissions, it is necessary to accurately distinguish *Cryptosporidium* spp. and *E. bieneusi* on the molecular level^10^. To date, at least 37 species and over 70 genotypes of *Cryptosporidium* spp. have been described^11^. Among them, 11 *Cryptosporidium* species have been identified in rodents, *Cryptosporidium parvum* and *C. muris* are the most common^12^. For *E. bieneusi*, more than 474 genotypes have been identified based on the internal transcribed spacer (ITS) region of the rRNA gene^13^, and more than 35 genotypes have been determined in rodents^14^. These genotypes can be classified into eleven groups (groups 1–11) by phylogenetic analyses^13^. Group 1 comprises the majority of zoonotic potential genotypes, whereas the remaining (groups 2–11) are considered as the host-adapted groups, which are mostly found in specific hosts or water^13^.

In China, *Cryptosporidium* spp. and *E. bieneusi* have been detected in a wide range of hosts, including carnivores, lagomorphs, primates, birds, and rodents^14,15^. Pet rodents, in particular (e.g. chinchillas, red-bellied tree squirrels, guinea pigs, and chipmunks), are considered potential sources of *Cryptosporidium* spp. and *E. bieneusi* infections in humans^16–19^. The red squirrel (*Sciurus vulgaris*) is a popular pet in China, which is widely bred in pet shops and homes for its appearance and mild-mannered nature. However, there is no published data regarding the prevalence of *Cryptosporidium* spp. and *E. bieneusi* in pet red squirrels, and the role of the red squirrels in the transmission of the two pathogens remains poorly investigated. Thus, we examined the occurrence of *Cryptosporidium* spp. and *E. bieneusi* in red squirrels, and evaluated their potential role in the zoonotic transmission of human cryptosporidiosis and microsporidiosis.

## Results

### Occurrence of *Cryptosporidium* spp. and *E. bieneusi*

The overall prevalence of *Cryptosporidium* spp. in pet red squirrels was 8.6% (27/314, 95% CI: 5.5–11.7%). All pet shops were positive for *Cryptosporidium*, and the prevalence ranged from 2.2% to 16.2%; significant differences were observed (χ^2^ = 0.028, df = 4, *P* < 0.05; Table 1). The prevalence of *Cryptosporidium* spp. among males and females were 8.1% and 9%, respectively, but the difference was not statistically significant (χ^2^ = 0.086, df = 1, *P* > 0.05). The differences in prevalence of *Cryptosporidium* spp. among squirrels of different ages were not statistically significant (χ^2^ = 0.093, df = 1, *P* > 0.05) (Table 2). Moreover, a significant correlation between the different sources and *Cryptosporidium* spp. infection (P = 0.01) was observed by logistic regression analysis.

**Table 1:** Occurrence of *Cryptosporidium* spp. and *Enterocytozoon bieneusi* in pet red squirrels from different sources in Southwestern China.

Sources	No. of examined	Cryptosporidium spp.				E. bieneusi			
		No. of positive	Prevalence (%) (95% CI)	OR (95% CI)	Species/Genotype (n)	No. of positive	Prevalence (%) (95% CI)	OR (95% CI)	Genotype (n)
Pet shop 1	58	2	3.4% (0.014–0.083)	reference	rat genotype I (2)	5	8.6% (0.012–0.161)	reference	D (3), RS01 (2)
Pet shop 2	74	12	16.2% (0.076–0.248)	5.4 (1.2–25.3)	rat genotype II (8), chipmunk genotype III (3), C. parvum (1)	16	21.6% (0.12–0.312)	2.9 (1.0–8.5)	D (6), SCC-2 (8), SCC-3 (2)
Pet shop 3	76	8	10.5% (0.035–0.176)	3.2 (0.7–16.1)	ferret genotype (6), chipmunk genotype III (2)	21	27.6% (0.173–0.379)	4.0 (1.4–11.5)	D (13), SCC-2 (6), RS02 (2)
Pet shop 4	61	4	6.6% (0.002–0.129)	2.0 (0.3–11.2)	rat genotype I (2), ferret genotype (2)	14	23% (0.121–0.338)	3.2 (1.1–9.4)	D (4), SCC-3 (10)
owners	45	1	2.2% (0.023–0.067)	0.6 (0.1–7.2)	C. parvum (1)	5	11.1% (0.016–0.207)	1.3 (0.4–4.9)	D (1), SCC-2 (4)
Total	314	27	8.6% (0.055–0.117)		rat genotype II (8), ferret genotype (8), chipmunk genotype III (5), rat genotype I (4), C. parvum (2)	61	19.4% (0.150–0.238)		D (27), SCC-2 (18), SCC-3 (12), RS01 (2), RS02 (2)

**Table 2:** Occurrence of *Cryptosporidium* spp. and *Enterocytozoon bieneusi* in pet red squirrels according to sex and age.

Factor	Characteristics	No. of examined	*Cryptosporidium spp*.			*E. bieneusi*		
			No. of positive	Prevalence (%) (95% CI)	OR (95% CI)	No. of positive	Prevalence (%) (95% CI)	OR (95% CI)
Sex	Male	148	12	8.1% (0.037–0.126)	reference	27	18.2% (0.119–0.245)	reference
	Female	166	15	9% (0.046–0.134)	1.1 (0.5–2.5)	34	20.5% (0.143–0.267)	1.2 (0.7–2.0)
Age	≤3 months	154	14	9.1% (0.045–0.137)	reference	32	20.8% (0.143–0.273)	reference
	>3 months	160	13	8.1% (0.038–0.124)	0.9 (0.4–1.9)	29	18.1% (0.121–0.242)	0.8 (0.5–1.5)

The overall prevalence of *E. bieneusi* in pet red squirrels was 19.4% (61/314, 95% CI: 15–23.8%). *E. bieneusi* was found in all four pet shops investigated, with infection rates ranging between 8.6% and 27.6%. The difference was statistically significant (χ^2^ = 0.036, df = 4, *P* < 0.05; Table 1). The prevalence of *E. bieneusi* in female red squirrels (20.5%) was higher than male (18.2%), but the difference was not statistically significant (χ^2^ = 0.251, df = 1, *P* > 0.05). The differences in prevalence of *E. bieneusi* among squirrels of different ages were not statistically significant (χ^2^ = 0.353, df = 1, *P* > 0.05) (Table 2). No mixed infections of the two pathogens were found in red squirrels in our study. Similarly, a significant correlation between the different sources and *E. bieneusi* infection (P = 0.01) was observed by logistic regression analysis.

### *Cryptosporidium* species/genotypes

**Figure 1:**
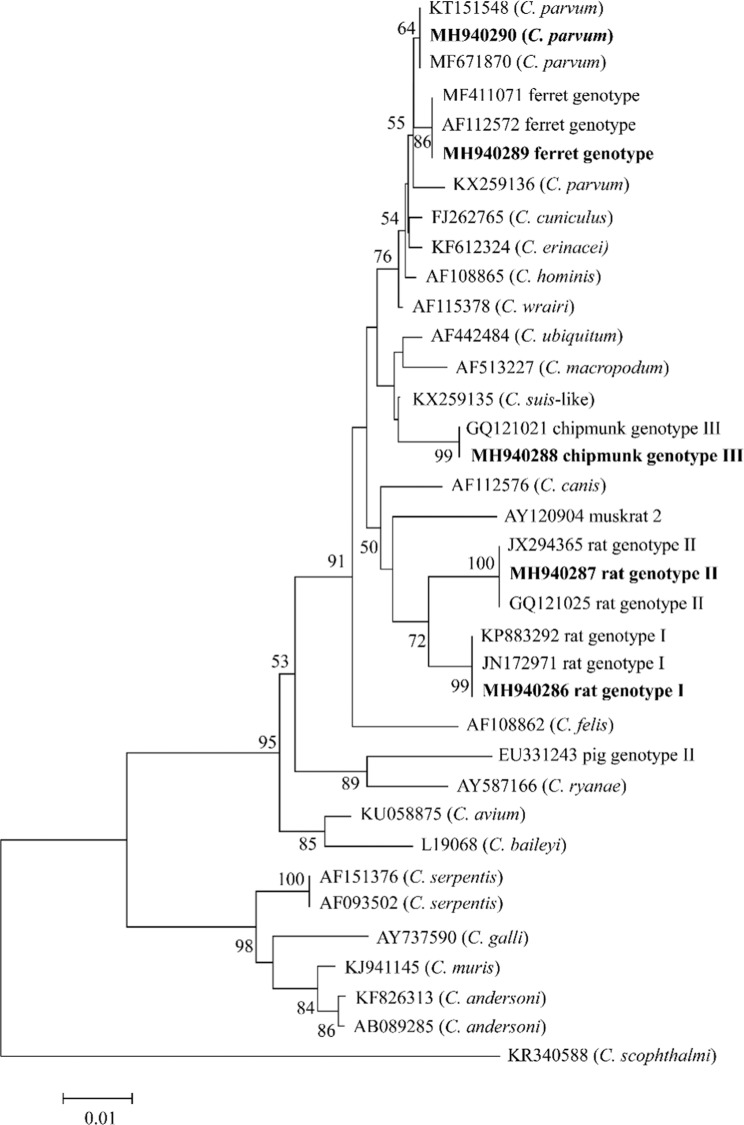
Phylogenetic relationships between the partial *Cryptosporidium* SSU rRNA gene from red squirrels and the *Cryptosporidium* spp. or genotypes deposited in GenBank. The GenBank accession number of each *Cryptosporidium* species or genotype is shown in parentheses. Bootstrap values above 50% from 1,000 replicates are shown at the nodes. The newly generated sequences are indicated in bold.

Twenty-seven *Cryptosporidium*-positive samples were genotyped by sequence analysis of the SSU rRNA gene, and five *Cryptosporidium* species/genotypes were identified: *Cryptosporidium* rat genotype II (8/27, 30%), *Cryptosporidium* ferret genotype (8/27, 30%), *Cryptosporidium* chipmunk genotype III (5/27, 18.5%), *Cryptosporidium* rat genotype I (4/27, 14.8%), and *C. parvum* (2/27, 7.4%). Phylogenetic relationship analysis confirmed the identity of *Cryptosporidium* species/genotypes (Fig. 1). *Cryptosporidium* rat genotype II and *Cryptosporidium* ferret genotype were the two most predominant genotypes (Table 1).

At the SSU rRNA locus, eight *Cryptosporidium* rat genotype II isolates had 100% homology between each other and were identical to that (GQ121025) from *Rattus tanezumi* in China and those from *R. rattus* in Australia (JX294365). The eight *Cryptosporidium* ferret genotype sequences were identical to reference sequences MF411071 (from *Sciurus vulgaris* in Italy) and AF112572 (from ferrets in the USA). *Cryptosporidium* chipmunk genotype III was identical to the reference sequence GQ121021 (from Siberian chipmunks in China); *Cryptosporidium* rat genotype I was identical to the reference sequences KP883292 (from *R. rattus* in Iran) and JN172971 (from *R. norvegicus* in Sweden); and *C. parvum* was identical to reference sequences MF671870 (from dairy cattle in China) and KT151548 (from coturnix in Iraq).

### *E. bieneusi* genotypes

DNA sequencing and subsequent analysis of the ITS-PCR products from the 61 *E. bieneusi*-positive specimens revealed the existence of three known *E. bieneusi* genotypes (D, SCC-2, SCC-4) and two novel genotypes, which were named RS01 and RS02. Genotype D was the most prevalent (44.3%, 27/61) and showed 100% homology with the sequences JF927954 (from humans in China) and AY371284 (from humans in Peru). Genotypes SCC-2 and SCC-4 had 100% homology with the two sequences MF410401 and MF410403, respectively.

With regard to the two novel genotypes, RS01 displayed two single nucleotide polymorphisms (SNPs) within the 243 bp of the ITS gene sequence of *E. bieneusi* (G/A at positions 178 and 324), when compared with the genotype SCC-2 (MF410401), which showed 99% homology. RS02 had three SNPs (G/A at positions 101 and 178, A/T at position 108) in comparison with genotype SCC-2, with 99% homology.

### Phylogenetic relationship of *E. bieneusi*

**Figure 2:**
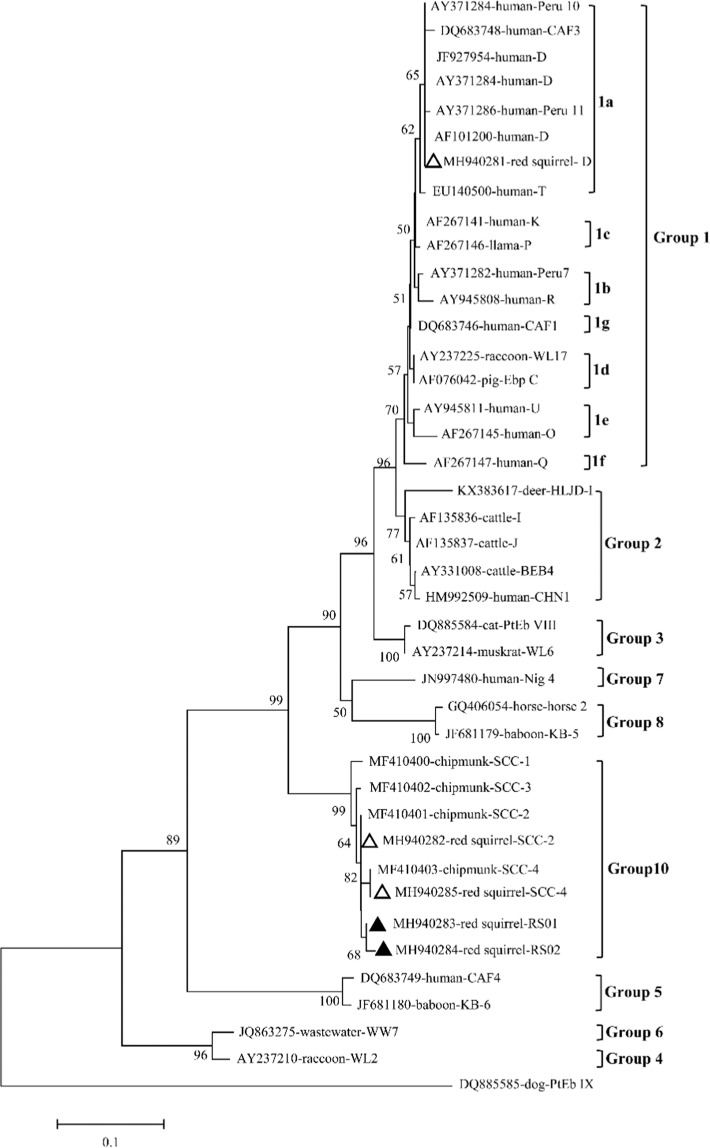
Phylogenetic relationships of the *E. bieneusi* genotypes identified in this study and other reported genotypes. The phylogeny was inferred with a neighbor-joining analysis of the internal transcribed spacer (ITS) sequences based on distances calculated with the Kimura two-parameter model. Bootstrap values greater than 50% from 1,000 replicates are shown at the nodes. Genotypes with open circles and solid circles are known and novel genotypes identified in this study, respectively.

A phylogenetic analysis of the ITS gene sequences of all the genotypes of *E. bieneusi* obtained here and reference genotypes published previously revealed that genotype D clustered in group 1 and further clustered into 1a, whereas genotypes SCC-2, SCC-4, and two novel genotypes (RS01 and RS02) clustered in group 10 (Fig. 2).

## Discussion

In 314 fecal samples of red squirrel, we first demonstrated the presence of *Cryptosporidium* spp. and *E. bieneusi*. The occurrence of *Cryptosporidium* spp. (8.6%, 27/314) was lower than the average prevalence previously reported in rodents (15.2%, 290/1911) (Table 3), but higher than those reported in laboratory rats in Nigeria (1.5%)^20^, brown rats in Iran (6.6%)^21^, and brown rats (6.2%), laboratory mice (1.7%), laboratory rats (4%), hamsters (7.8%), squirrel monkeys (4.2%), and Bamboo rats (3.3%) in China^22–24^. *Cryptosporidium* spp. has been reported in multiple rodent species, with an infection rate of 1.5% in laboratory rats to 85.0% in guinea pigs^16,25–27^. The relatively low occurrence of *Cryptosporidium* spp. in this study may be explained by the fact that the pet squirrels lived in clean environments and were kept in separate cages. The prevalence of *E. bieneusi* was 19.4%, which was similar to that in two recent studies in Sichuan province for *E. bieneusi* infection rates in red-bellied tree squirrels (16.7%, 24/144)^18^ and chipmunks (17.6%, 49/279)^17^. The prevalence of *E. bieneusi* in rodents ranged from 1.1% to 100% (Table 4)^12,19,28–30^. As proposed in other studies, factors contributing to the prevalence of these pathogens may include the examination method, age, sex, season, host health status, feeding density, sample size, geo-ecological conditions, and living conditions^31–33^.

**Table 3:** Occurrence of *Cryptosporidium* species/genotypes in rodents from different countries.

Country	Host (common name)	Scientific name	No. of samples	No. of positive (%)	Species/Genotype (n)	References
Japan	Brown rats	*Rattus norvegicus*	50	19 (38)	*C. meleagridis* (1), *C. parvum* (1), New genotypes (11)	Kimura *et al*., 2007
USA	Opossum	*Didelphis virginiana*	—	2	Marsupial genotype (2)	Feng *et al*., 2007
	Chipmunk	*Tamias striatus*	—	1	*C. baylei* (1)	
	Gray squirrel	*Sciurus carolinensis*	—	1	Skunk genotype (1)	
	White-footed mouse	*Peromyscus leucopus*	—	3	*C. parvum* (3)	
	Deer mouse	*Peromyscus maniculatus*	—	3	*C. parvum* (2), Muskrat II genotype (1)	
	Red-backed vole	*Clethrionomys gapperi*	—	2	*C. parvum* (1), Muskrat II genotype (1)	
	Meadow vole	*Microtus pennsylvanicus*	—	5	Muskrat II genotype (5)	
	House mouse	*Mus musculus*	—	1	*C. parvum* (1)	
Australia	Black rats	*Rattus rattus*	85	7 (8.2)	rat genotype III (4), rat genotype II (3)	Koehler *et al*., 2018
	Swamp rats	*Rattus lutreolus*	21	3 (14.3)	C. viatorum (3)	
Philippines	Asian house rat	*Rattus tanezumi*	83	37 (44.6)	rat genotype III (18), rat genotype IV (5), *suis*-like genotype (5), *C. scrofarum* (3), rat genotype I (1), rat genotype II (1), *C. muris* (1)	Nghublin *et al*., 2013
	Brown rat	*Rattus norvegicus*	70	12 (18.6)	rat genotype II (5), rat genotype IV (1), *C. muris* (2), rat genotype I (2), *C. scrofarum* (1), rat genotype III (1)	
Iran	Brown rat	*Rattus norvegicus*	91	6 (6.6)	*C. parvum* + *C. muris* (6)	Gholipoury *et al*., 2016
Nigeria	Laboratory rats	*Rattus norvegicus*	134	2 (1.5)	*C. andersoni* (1), rat genotype II (1)	Ayinmode *et al*., 2017
Slovak Republic	Striped field mouse	*Apodemus agrarius*	103	34 (33)	*C. scrofarum* (18), *C. parvum* (9), Muskrat genotype II (3), *C*. environment isolate (3), *C. hominis* (1)	Danišová *et al*., 2017
	Bank vole	*Myodes glareolus*	72	16 (22.2)	*C. scrofarum* (4), *C. parvum* (3), Muskrat genotype I (3), *C. environment isolate* (6)	
	Yellow-necked mouse	*Apodemus flavicollis*	73	15 (20.5)	*C. scrofarum* (5), *C. suis* (4), *C. parvum* (3), *C*. environment isolate (3)	
Italy	Red squirrels	*Sciurus vulgaris*	70	17 (24.3)	ferret genotype (15), chipmunk genotype I (2)	Kvác *et al*., 2008
China	Brown rat	*Rattus norvegicus*	64	4 (6.2)	*C. tyzzeri* (3), rat genotype III (1)	Lv *et al*., 2009
	Asian house rat	*Rattus tanezumi*	33	5 (15.2)	*C. tyzzeri* (1), rat genotype II (2), rat genotype III (2)	
	Laboratory mouse	*Mus musculus*	229	4 (1.7)	*C. tyzzeri* (4)	
	Laboratory rat	*Rattus norvegicus*	25	1 (4)	*C. tyzzeri* (1)	
	Golden hamster	*Mesocricetus auratus*	50	16 (32)	*C. muris* (7), *C. andersoni* (5), *C. parvum* (4)	
	Siberian hamster	*Phodopus sungorus*	51	4 (7.8)	*C. parvum* (2), *C. muris* (1), hamster genotype (1)	
	Campbell hamster	*Phodopus campbelli*	30	3 (10)	*C. parvum* (2), *C. andersoni* (1)	
	Red squirrel	*Sciurus vulgaris*	19	5 (26.3)	ferret genotype (5)	
	Siberian chipmunk	*Tamias sibiricus*	20	6 (30)	ferret genotype (4), *C. parvum* (1), *C. muris* (1), chipmunk genotype III (1)	
	Guinea pig	*Cavia porcellus*	40	34 (85)	*C. wrairi* (30)	
	Chinchillas	*Chinchilla lanigera*	140	14 (10)	*C. ubiquitum* (13), *C. parvum* (1).	Qi *et al*., 2015
	Brown rats	*Rattus norvegicus*	242	22 (9.1)	rat genotype I (14), rat genotype IV (6), *C. suis*-like genotype (1), *C. ubiquitum* (1)	Zhao *et al*., 2018
	Squirrel monkey	*Saimiri sciureus*	24	1 (4.2)	*C. hominis* monkey genotype II (1)	Liu *et al*., 2015a
	Bamboo rats	*Rhizomys sinensis*	92	3 (3.3)	*C. parvum* (3)	Liu *et al*., 2015b

-represents unknown.

**Table 4:** Occurrence and genotypes of *Enterocytozoon bieneusi* in rodents from different countries.

Country	Host (common name)	Scientific name	No. of samples	No. of positive (%)	Genotypes (no.)	References
Czech Republic and Germany	East-European house mice	*Mus musculus musculus*	127	14 (11)	D (6), PigEBITS5 (4), EpbA (2), C (1), H (1)	Sak *et al*., 2011
	West-European house mice	*Mus musculus domesticus*	162	17 (10.5)	D (4), Peru 8 (4), CZ3 (4), PigEBITS5 (3), S6 (1), C (1)	
United States	Eastern gray squirrel	*Sciurus carolinensis*	34	11 (32.4)	WL4 (5), Type IV (3), WW6 (2), PtEbV + WL21 (1)	Guo *et al*., 2014
	Eastern chipmunk	*Tamias striatus*	7	5 (71.4)	WL4 (3), Type IV (1), WL23 (1)	
	Woodchuck	*Marmota monax*	5	5 (100)	WL4 (2), Type IV + WL20 (1), WL22 (1), WW6 (1)	
	Deer mouse	*Peromyscus sp*.	55	13 (23.6)	WL4 (10), WL23 (2), WL25 (1)	
	Boreal red-backed vole	*Myodes gapperi*	5	1 (20)	WL20 + WL21(1)	
	Meadow vole	*Microtus pennsylvanicus*	10	3(33)	Peru11 (1), Peru11 + type IV (1), WL21 + unknown (1)	
	Guinea pigs	*Cavia porcellus*	60	4 (6.7)	Peru 16 (4)	Cama *et al*., 2007
	Black-tailed prairie dogs	*Cynomys ludovicianus*	153	14 (9.2)	Row (14)	
Poland	Pallas	*Apodemus agrarius*	184	79^a^	D (6), WR8 (2), WR5 (1), WR7 (1), gorilla 1 (1)	Perec-Matysiak *et al*., 2015
	Yellow-necked mouse	*Apodemus flavicollis*	60	18^a^	D (2), WR6 (6), WR4 (1), WR1 (1), WR9 (1)	
	Bank vole	*Myodes glareolus*	46	18^a^	D (2), WR6 (2), WR10 (2), WR2 (1)	
	House mouse	*Mus musculus*	21	6^a^	WR3 (1)	
Slovakia	House mouse	*Mus musculus musculus*	280	3 (1.1)	Unknown	Danišová *et al*., 2015
China	Chinchillas	*Chinchilla lanigera*	140	5 (3.6)	D (2), BEB6 (3)	Qi *et al*.,
	Brown rats	*Rattus norvegicus*	242	19 (7.9)	D (17), Peru6 (2)	Zhao *et al*., 2018
	Red-bellied tree squirrels	*Callosciurus erythraeus*	144	24 (16.7)	D (18), EbpC (3), SC02 (1), CE01 (1), CE02 (1)	Deng *et al*., 2016
	Chipmunks	*Eutamias asiaticus*	279	49 (17.6)	D (6), SCC-1 (17), SCC-2 (9), SCC-3 (5), CHY1 (5), Nig 7 (4), CHG9 (2), SCC-4 (1)	Deng *et al*., 2018a

^a^Represents positive samples in feces and spleen.

Previous studies have indicated that five *Cryptosporidium* species and nine *Cryptosporidium* genotypes exist in various rodents in China (Table 3)^12,22,23^. In this study, five different *Cryptosporidium* species/genotypes were identified, including *Cryptosporidium* rat genotypes I and II, *Cryptosporidium* ferret genotype, *Cryptosporidium* chipmunk genotype III, and *C. parvum*. *Cryptosporidium* rat genotypes I and II have been found in brown rats in the Philippines^34^, Nigeria^20^, Australia^35^, and China^22^, even in South Nation River watershed, raw wastewater, and environmental samples in the United Kingdom, Canada, and China^36–38^. *Cryptosporidium* ferret genotype has been found in ferrets and red squirrels in Italy^39^. The *Cryptosporidium* chipmunk genotype III was previously reported in red squirrels, eastern squirrels, eastern chipmunks, and deer mice in the USA^40^. To date, little is known regarding the disease-causing potential of the four genotypes in humans and livestock; thus bringing attention to the need for epidemiological molecular surveillance of *Cryptosporidium* spp. for the assessment of infectivity across different hosts.

*C. parvum* is one of the two predominant *Cryptosporidium* species in humans^41^. *C. parvum* has been identified in humans in Henan province, China^42^. Moreover, *C. parvum* infections have been observed in brown rats in Japan, mice and red-backed voles in the USA, brown rats in Iran, striped field mice in Slovak Republic, and hamsters, Siberian chipmunks, chinchillas, and Bamboo rats in China, highlighting the prevalence of *C. parvum* in rodents (Table 3)^12,25,27^. In addition, *C. parvum* has also been found in other animals, such as cattle, sheep, goats, deer, alpacas, horses, dogs, gray wolves, raccoon dogs, cats, and pigs^43,44^. In this study, only two *C. parvum* isolates were identified in investigated red squirrels; however, these isolates may result in emerging zoonotic infections through the oral-fecal route.

Three known genotypes (D, SCC-2, and SCC-3) and two novel genotypes (RS01 and RS02) were identified in this study. Genotype D was the predominant genotype (44.3%, 27/61). This finding was similar to previous reports in mice in Czech Republic and Germany^45^ (32.3%, 10/31), mice in Poland^46^ (33.3%, 10/30), and brown rats (89.5%, 17/19) and red-bellied tree squirrels (75%, 18/24) in China^12,18^. In China, genotype D has been identified in humans and various animals, such as nonhuman primates, cattle, sheep, horses, pigs, dogs, cats, and in wastewater^14,47–49^. This study demonstrated the presence of genotype D in red squirrels for the first time, suggesting that red squirrels could play a potential role in the disease dissemination of *E. bieneusi* to humans.

## Conclusions

This is the first report on the incidence of *Cryptosporidium* spp. and *E. bieneusi* in pet red squirrels in China. The infection rates of *Cryptosporidium* spp. and *E. bieneusi* were 8.6% and 19.4%, respectively. The detection of zoonotic *C. parvum* and genotype D of *E. bieneusi* suggests that red squirrels are a potential source of cryptosporidiosis and microsporidiosis in humans. However, the infection sources and transmission dynamics between red squirrels and humans remain unknown, thus emphasizing on the importance of further follow-up studies of the transmission dynamics of these pathogens.

## Materials and Methods

### Ethics statement

The present study protocol was reviewed and approved by the Research Ethics Committee and the Animal Ethical Committee of Sichuan Agricultural University, and all methods were performed in accordance with the relevant guidelines and regulations. Permission was obtained from the owners or shop managers before the fecal specimens were collected.

### Collection of specimens

A total of 314 fecal specimens were collected from red squirrels from four pet shops (n = 269) and owners (n = 45) in the Sichuan province, southwestern China between September 2016 and December 2017 (Table 1). All tested pet shops only raised red squirrels and served as suppliers of red squirrels to other pet shops. Sample size was approximately 20% of the squirrels from each shop, and small-scale shops (population less than 50) were not included. The four pet shops are distributed in Jianyang (104°32′E, 30°24′N), Pengzhou (103°57′E, 30°59′N), Wenjiang (103°51′E, 30°40′N), and Jintang (104°24′E, 30°51′N). Pet squirrels from owners were primarily distributed around the urban areas of Chengdu city (104°03′E–104°08′E, 30°36′N–30°52N′). In both pet shops and homes, red squirrels were housed in separate cages. Approximately 30–50 g fresh fecal samples were collected from the bottom of each cage after defecation using a sterile disposal latex glove and then immediately placed into individual disposable plastic bags. No obvious clinical signs were observed at the time of sampling, and the age, sex, and source were recorded at the same time. All fecal specimens were stored in 2.5% potassium dichromate solution at 4 °C until processing.

### DNA extraction

The fecal specimens were washed three times in distilled water with centrifugation at 3,000 × *g* for 10 min to remove the potassium dichromate. Genomic DNA was extracted from approximately 200 mg of each processed fecal specimen using an E.Z. N. A. R Stool DNA kit (Omega Biotek Inc., Norcross, GA, USA) according to the manufacturer’s recommended instructions. The extracted DNA was stored at −20 °C until molecular analysis.

### Genotyping of *Cryptosporidium* spp. and *E. bieneusi*

*Cryptosporidium* spp. were identified by nested polymerase chain reaction (PCR) amplification of an SSU rRNA gene fragment of ~830 bp designed by Xiao *et al*.^50^. *E. bieneusi* genotypes were determined by nested PCR amplification of a 392-bp fragment containing the entire ITS (243 bp) and portions of the flanking large and small subunits of the rRNA gene^31^ (Supplementary Table 1). TaKaRa Taq DNA Polymerase (TaKaRa Bio, Otsu, Japan) was used for PCR amplification. Positive controls (camel-derived *C. andersoni* DNA for *Cryptosporidium* spp. and horse-derived genotype D DNA for *E. bieneusi*) and negative control with no DNA added were included in all PCR assays. The secondary PCR products were examined by agarose gel electrophoresis and visualized after ethidium bromide staining.

### Sequence analysis

All nested PCR positive-products were sequenced using the same PCR primers as those used for the secondary PCRs on an ABI 3730 instrument (Applied Biosystems, Foster City, CA, USA) at the BioSune Biotechnology Company (Shanghai, China). The nucleotide sequences of each obtained gene were aligned and analyzed using the Basic Local Alignment Search Tool and Clustal X (http://www.clustal.org/) with reference sequences retrieved from GenBank to identify *Cryptosporidium* spp. and *E. bieneusi* genotypes.

### Phylogenetic analyses

To support the *Cryptosporidium* species/genotypes and assess the genetic relationships between the *E. bieneusi* genotypes in the present study and reference sequences previously published in GenBank, phylogenetic analysis was performed using Phylip version 3.69 package and by constructing a neighboring-joining tree using Mega 6 software (http://www.megasoftware.net/), which is based on evolutionary distances calculated using a Kimura 2-parameter model. The MegAlign program in the DNA Star software package (version 5.0) was used to determine the degree of sequence identity. The reliability of these trees was assessed using bootstrap analysis with 1,000 replicates.

### Statistical analysis

Variations in the occurrence of *Cryptosporidium* spp. (y1) and *E. bieneusi* (y2) in red squirrels according to age (x1), sex (x2), and geographical location (x3) were analyzed by χ^2^ test using SPSS V20.0 (IBM, Chicago, IL, USA). Each of these variables was included in the binary logit model as an independent variable by multivariable regression analysis. When the *P* value was less than 0.05, the results were considered statistically significant. The adjusted odds ratio (OR) and 95% confidence interval (CI) for each variable were calculated with binary logistic regression, and all risk factors were entered simultaneously.

### GenBank accession numbers

Representative nucleotide sequences were deposited in GenBank with the following accession numbers: MH940281-MH940290.

## Supplementary information

Supplementary information

## Data Availability

All data generated or analysed during this study are included in this published article and its Supplementary Information Files.
