# Supplementary material for: Occurrence and genetic characteristics of Cryptosporidium spp. and Enterocytozoon bieneusi in pet red squirrels (Sciurus vulgaris) in China
Source: Sci Rep. 2020 Jan 23;10:1026. doi: 10.1038/s41598-020-57896-w (PMC6978461; doi:10.1038/s41598-020-57896-w)
Supplement: Supplementary file 1 — Supplementary information [file 41598_2020_57896_MOESM1_ESM.docx]

**Supplementary Information**

Occurrence and genetic characteristics of *Cryptosporidium* spp. and *Enterocytozoon bieneusi* in pet red squirrels (*Sciurus vulgaris*) in China

Lei Deng^1^, Yijun Chai^1^, Run Luo^1^, Leli Yang^1^, Jingxin Yao^1^, Zhijun Zhong^1^, Wuyou Wang^1^, Leiqiong Xiang^1^, Hualin Fu^1^, Haifeng Liu^1^, Ziyao Zhou^1^, Chanjuan Yue^2^, Weigang Chen^2^, Guangneng Peng^1^*

*^1^The Key Laboratory of Animal Disease and Human Health of Sichuan Province, College of Veterinary Medicine, Sichuan Agricultural University, Chengdu, Sichuan, 611130, China.*

*^2^Chengdu Research Base of Giant Panda Breeding, Sichuan Key Laboratory of Conservation Biology for Endangered Wildlife, Sichuan Academy of Giant Panda, Chengdu, Sichuan Province 611130, China.*

* To whom correspondence should be addressed. E-mail: pgn.sicau@163.com

**Supplementary Table 1. Primer sequences and annealing temperatures of the genes used in this study, as well as the fragment lengths of the PCR products.**

| Gene | Primer | Sequence (5'-3') | Annealing temperature (°C) | Fragment length (bp) | Reference |
| --- | --- | --- | --- | --- | --- |
| SSU rRNA | F1 | CCCATTTCCTTCGAAACAGGA | 55 | 830 | Xiao, L. *et al*. |
|  | R1 | TTCTAGAGCTAATACATGCG |  |  |  |
|  | F2 | AAGGAGTAAGGAACAACCTCCA | 58 |  |  |
|  | R2 | GGAAGGGTTGTATTATTAGATAAAG |  |  |  |
| ITS | F1 | GATGGTCATAGGGATGAAGAGCTT | 55 | 392 | Sulaiman, I. M. *et al*. |
|  | R1 | AATACAGGATCACTTGGATCCGT |  |  |  |
|  | F2 | AGGGATGAAGAGCTTCGGCTCTG | 55 |  |  |
|  | R2 | AATATCCCTAATACAGGATCACT |  |  |  |

Xiao, L. *et al.* Phylogenetic analysis of *Cryptosporidium* parasites based on the small-subunit rRNA gene locus. *Appl Environ Microbiol* **65**, 1578 (1999).

Sulaiman, I. M. *et al.* Molecular Characterization of Microsporidia Indicates that Wild Mammals Harbor Host-Adapted *Enterocytozoon* spp. as well as Human-Pathogenic *Enterocytozoon bieneusi*. *Appl Environ Microbiol* **69**, 4495 (2003).
